# Husband involvement in antenatal care moderates the link between vitamin D status and depressive symptoms in pregnant women

**DOI:** 10.1017/S2045796025000022

**Published:** 2025-02-12

**Authors:** Rosa S. Wong, Keith T. S. Tung, Hing Wai Tsang, Jennifer K. Y. Ko, Wing-cheong Leung, Patrick Ip

**Affiliations:** 1 Department of Special Education and Counselling, The Education University of Hong Kong, Hong Kong SAR, China; 2 Department of Paediatrics and Adolescent Medicine, The University of Hong Kong, Hong Kong SAR, China; 3 Department of Obstetrics and Gynaecology, The University of Hong Kong, Hong Kong SAR, China; 4 Department of Obstetrics and Gynaecology, Kwong Wah Hospital, Hong Kong SAR, China

**Keywords:** husband involvement in antenatal care, pregnancy, serum 25(OH)D concentration, social support, vitamin D status

## Abstract

**Aims:**

The association between a pregnant mother’s vitamin D status and depressive symptoms has yielded inconsistent results. It is possible that other factors play a role in this association, as depression can have multiple causes. Recognizing the significance of the husband’s participation in antenatal care, this study aimed to examine whether the husband’s involvement moderates the link between the mother’s vitamin D status and depressive symptoms during pregnancy.

**Methods:**

A total of 2983 Chinese married pregnant women, in their 25–35 weeks of pregnancy, completed questionnaires to assess their levels of depressive symptoms and the involvement of their husbands in their antenatal care appointments. Additionally, their serum levels of vitamin D were measured.

**Results:**

After adjusting for maternal age, parity, and socio-economic status, the husband’s involvement in antenatal care moderated the association between maternal vitamin D status and depressive symptoms during pregnancy (β = 2.03, *p* = 0.035). Specifically, when their husbands were not regularly present for antenatal care appointments, mothers with suboptimal vitamin D levels experienced more depressive symptoms than those with optimal levels. However, there were no noticeable differences in depressive symptoms between vitamin D groups for mothers whose husbands attended all antenatal care appointments.

**Conclusions:**

Pregnant women who have suboptimal vitamin D levels and lack support from their spouses are most vulnerable to experiencing depression. It is crucial to holistically assess the social and physiological needs of expectant mothers to reduce their risk of antenatal depression.

## Introduction

Pregnancy is a time of both extreme joy and significant stress. Studies show that pregnant women are particularly susceptible to depression, with a prevalence rate ranging from 6% to 20% (Biaggi *et al.*, 2016; Woody *et al.*, 2017). Even if they do not meet the criteria for a clinical diagnosis of depression, experiencing elevated depressive symptoms can result in poor mental health and overall well-being, as well as impact the well-being of their offspring during and after pregnancy (Sethna *et al.*, 2021; Zhang *et al.*, 2023). The causes of depression during pregnancy are not fully understood, but there is growing evidence indicating the complex aetiology of this neuropsychiatric condition, which can be influenced by a combination of biological, cognitive and psychosocial factors. Specifically, biological factors pertain to how the body reacts to stress (Nedic Erjavec *et al.*, 2021), cognitive factors relate to how information is processed (Kube *et al.*, 2020), and psychosocial factors concern the level of social support and psychological resources available to cope with stress (Gariépy *et al.*, 2016). Over time, negative experiences with these factors may contribute to a ‘negative cognitive triad’, characterized by negative beliefs about oneself, the world and the future. These beliefs can further exacerbate cognitive biases and stress responses, ultimately contributing to the development of depressive symptoms (Beck and Bredemeier, 2016).

From a biological and neurocognitive standpoint, elevated cortisol levels during pregnancy could increase the likelihood of depression in pregnant women by disrupting the hypothalamic–pituitary–adrenal axis and reducing sensitivity of glucocorticoid receptors in the brain (Francisco Juruena *et al.*, 2015; Swales *et al.*, 2018). However, protective and risk factors may interact to reduce the risk of depression during pregnancy. One factor contributing to individual differences in neurocognitive functions is vitamin D, which has been suggested as a possible solution to counteract the negative effects of glucocorticoids on mood and cognitive function and improve synaptic function in the hippocampus (Lardner, 2015). The wide distribution of vitamin D receptors and activating enzyme 1-α-hydroxylase (CYP27B1) throughout the brain regions may also contribute to the connection between vitamin D and neuropsychiatric disorders (Eyles, 2021). Recent studies have shown that both the circulating and biologically active forms of vitamin D (i.e., 25(OH)D and 1,25(OH)_2_D) can penetrate the blood–brain barrier (Mayne and Burne, 2019; Sangha *et al.*, 2023), thereby prompting researchers to investigate its potential neuroprotective effects and its possible association with depression. Previous systematic reviews and meta-analyses have produced mixed results concerning the relationship among circulating 25(OH)D levels, vitamin D supplementation, and depression (Gowda *et al.*, 2015; Vellekkatt and Menon, 2019). A recent meta-analysis revealed a significant link between vitamin D supplementation and a reduction in depressive symptoms (Mikola *et al.*, 2022), whereas a systematic review documented some studies reporting no association (Aghajafari *et al.*, 2018). Further research is necessary to fully understand the complex relationship between vitamin D and depression.

In addition to variations in methodology across studies, individual factors also play a role in the onset of depression during pregnancy. While biological and neurocognitive factors are important, the psychosocial perspective has gained prominence, particularly with regard to the influence of spousal support and involvement in antenatal care on pregnant women’s susceptibility to depression. A meta-analysis revealed a significant correlation between inadequate social support and antenatal depression, which was corroborated by qualitative reviews of 14 out of 15 studies (Bedaso *et al.* 2021). Previous research investigated how the size and composition of a pregnant woman’s supportive network impacted the risk of depression and found that a larger non-family supportive network was associated with a decreased risk, but no association was found with the size of the family supportive network (Friedman *et al.*, 2020). These results suggest that the specific identities of family members could have an impact on depression risk, even though the number of available family members may have no impact. A population-based study found that mothers who received social support solely from their spouse had a lower likelihood of developing postpartum depression than those who received support from other sources (Yamada *et al.*, 2020). These results are consistent with the accounts of women in a focus group interview who reported experiencing negative emotions towards their marriage and couple relationship due to their husband’s inconsistent involvement during pregnancy (Eddy and Fife, 2021). It is thus important to assess the extent of husband involvement when examining the causes of maternal depressive symptoms during pregnancy.

Although many studies have investigated the causes of depression, few have explored the interplay between biological and psychosocial factors. This is particularly critical during pregnancy, as the emotional and physiological changes that occur can have a combined impact on how expectant mothers perceive their pregnancy. Hence, the main objective of this study is to expand our understanding of individual differences in the association between vitamin D status and depressive symptoms among pregnant women. Bowen’s theory emphasizes the interconnectedness of individuals within their relationships and proposes that emotional well-being is influenced by and influences others (Bowen, 1972, 1976). Since each person has a unique set of psychosocial experiences and perceptions, the degree of psychological protection gained from a husband’s involvement in antenatal care could differ among pregnant women, potentially strengthening or weakening the relationship between vitamin D levels and depressive symptoms. For example, some women may have optimal vitamin D levels but receive inadequate support from their partners during pregnancy, while others may receive adequate support but have suboptimal vitamin D levels. Based on these theoretical assumptions, we hypothesized that the extent of husband involvement in antenatal care would moderate the association between vitamin D status and depression levels in pregnant women. Specifically, suboptimal vitamin D status would be linked to depressive symptoms in women with inactive husband involvement, while this association would not be observed in women with active husband involvement.

## Method

### Study design and participants

This cross-sectional study recruited a total of 2983 Chinese expectant mothers from antenatal care clinics in Hong Kong. Power analysis conducted in G*Power 3.1 (Faul *et al.*, 2009) indicated that this sample size would allow for the detection of a very small effect of partial *R*^2^ increase of 0.004 (α = 0.05) with a power of 0.84. The mothers did not receive any form of compensation for participating in the study.

### Procedure

Expectant mothers in their 25–35 weeks of pregnancy were recruited from the waiting area of the antenatal clinic. After obtaining written informed consent, participants filled out questionnaires regarding their demographics, their husband’s involvement in antenatal care, and their own depressive symptoms. Trained phlebotomists also collected peripheral blood samples from the participants. This study was approved by the Institutional Review Board of the University of Hong Kong/Hospital Authority Hong Kong West Cluster Research Ethics Committee (UW 13-055). All participants provided informed consent to participate in the study.

### Measures

#### Depressive symptoms

Depressive symptoms were assessed with the Chinese version of the 20-item Center for Epidemiologic Studies Depression Scale (CES-D) (Radloff, 1977). Using a scale from 0 = rarely or none of the time to 3 = most or all of the time, participants rated the frequency of their cognitive, affective and somatic depressive symptoms. The scores of individual items were added together to calculate a total score, which ranges from 0 (lowest) to 60 (highest). These scores were classified into four groups: not depressed (0–9 points), mildly depressed (10–15 points), moderately depressed (16–24 points) and severely depressed (more than 25 points). For the purposes of this study, participants were further classified as likely to have clinical depression (CES-D ≥ 16) or unlikely to have clinical depression (CES-D < 16). The CES-D has shown strong reliability and validity (Radloff, 1977), and in this particular sample, its internal reliability was high (α = 0.90).

#### Serum 25(OH)D concentrations

The serum was isolated from the collected peripheral blood samples. For this study, the liquid chromatography-tandem mass spectrometry method was utilized to determine the concentration of serum 25(OH)D. This method involves the measurement of 25(OH)D3 and 25(OH)D2, minus 3-Epi-25(OH)D3, using the QTRAP 5500 LC-MS/MS system (AB SCIEX Instruments, Framingham, MA, USA). Serum 25(OH)D concentrations can be used to categorize participants into three groups: sufficient vitamin D (25[OH]D concentrations ≥ 50 nmol/L), insufficient vitamin D (25 nmol/L ≤ 25[OH]D concentrations < 50 nmol/L), and deficient vitamin D (25[OH]D concentrations < 25 nmol/L). For the purposes of this study, participants were further categorized as having optimal vitamin D status (25[OH]D concentrations ≥ 50 nmol/L) or having suboptimal vitamin D status (25[OH]D concentrations < 50 nmol/L). The accuracy of this method was assessed against samples from the Vitamin D External Quality Assessment Scheme, and it demonstrated satisfactory performance within ±15% of the target value (Carter *et al.*, 2017).

#### Husband involvement in antenatal care

The level of husband’s participation in antenatal care was assessed by asking the participant whether her husband had accompanied her to three types of antenatal appointments: pre-pregnancy check-ups, courses and ultrasound sessions. Participants who responded ‘Yes’ to all three questions were classified as having husbands who were actively involved in antenatal care, while those who did not were classified as having husbands who were less involved in antenatal care.

#### Covariates

In addition to the participant’s age and parity (i.e., the number of live births and stillbirths at viable gestational age), a composite socio-economic index was created to give a more precise classification of the participant’s socio-economic position within the sample. Specifically, the index was computed by normalizing and aggregating all the relevant socio-economic indicators including monthly family income adjusted for household size and the education level and employment status of the participant and her husband.

### Data analysis

IBM SPSS version 27 for Windows was used to conduct all the analyses. To test our primary hypothesis, we utilized regression analysis with bootstrapping methods, and Andrew F. Hayes PROCESS 3.2.01 macro was employed for this purpose (Hayes, 2017).

## Results

All mothers were married and between the ages of 17–43 years (*M* = 33.49, *SD* = 4.19). 167 mothers (5.6%) did not graduate from high school, 1056 mothers (35.4%) reported having completed secondary education, and 1760 mothers (59%) reported having completed higher education. Most of the participants were employed full time (*N* = 2192, 73.5%). Additionally, 1266 mothers (42.4%) had prior childbirth experience. The results of the CES-D frequency analysis indicated that 1057 (35.4%) had no depressive symptoms and 1453 (48.7%) were mildly depressed, whereas the rest of the sample displayed moderate (*N* = 352, 11.8%) or severe (*N* = 121, 4.1%) depressive symptoms. Regarding their vitamin D status, 2716 (91%) had serum 25(OH)D concentrations that were within the optimal range (≥50 nmol/L), while 255 (8.5%) had insufficient concentrations (25–49 nmol/L) and 12 (0.4%) had deficient concentrations (<25 nmol/L). Among the 2983 expectant mothers, 956 (32%) reported that their husband accompanied them to every antenatal care appointment, including pre-pregnancy check-ups, courses and ultrasounds. On the other hand, the husbands of the remaining participants were only present for certain appointments or not at all.

Subsequently, we examined descriptive statistics, conducted correlation analysis, and assessed variations in age, parity, monthly household income adjusted for household size, depression scores and levels of husband involvement in antenatal care among participants with suboptimal 25(OH)D concentrations (below 50 nmol/L) and those with optimal concentrations. Table 1 shows the results of these analyses. Specifically, the level of husband involvement in antenatal care did not differ by maternal vitamin D status. However, mothers with optimal 25(OH)D concentrations were found to be older, have fewer childbirth experiences, and have a higher family income compared to those with suboptimal 25(OH)D concentrations. In addition, mothers with optimal 25(OH)D concentrations had few depressive symptoms, while those with suboptimal 25(OH)D concentrations had scores falling within the range for mild depressive symptoms. Furthermore, all the variables of interest were significantly interrelated. Noteworthy is the positive, albeit weak, correlation between parity and depressive symptoms, as well as the negative correlation between parity and husband involvement. Higher family income was linked to lower depression scores and higher levels of husband involvement, whereas a weak negative correlation was found between depressive symptoms and husband involvement.

**Table 1.** Pearson’s correlations between variables, means, standard deviations in the sample and within optimal and suboptimal vitamin D groups and results of t-test of vitamin D group differences in variables

	Age	Parity	Adjusted monthly family income (HKD)	Depressive symptoms	Husband involvement
Age	–				
Parity	0.22**	–			
Adjusted monthly family income	0.07**	−0.32**	–		
Depressive symptoms	−0.08**	0.04*	−0.14**	–	
Husband involvement	−0.05**	−0.26**	0.20**	−0.09**	–
*M* general	33.49	0.53	21,941.46	8.66	2.16
*SD* general	4.19	0.71	16,727.04	6.16	0.74
*M* suboptimal 25(OH)D	32.85	0.75	17,209.77	9.66	2.12
*SD* suboptimal 25(OH)D	4.66	0.91	14,063.50	8.21	0.82
*M* optimal 25(OH)D	33.56	0.51	22,406.62	8.56	2.17
*SD* optimal 25(OH)D	4.13	0.69	16,897.15	7.04	0.73
*t* (2981)	−2.41	4.24	−5.65	2.12	−1.02
*p*	0.017	<0.001	<0.001	0.04	0.31
Hedge’s *g*	0.17	0.34	0.31	0.15	0.07

Hedge’s *g* was employed as it tackles unevenness in sample sizes.

* *p* < 0.05.

** *p* < 0.001.

**Figure 1.**
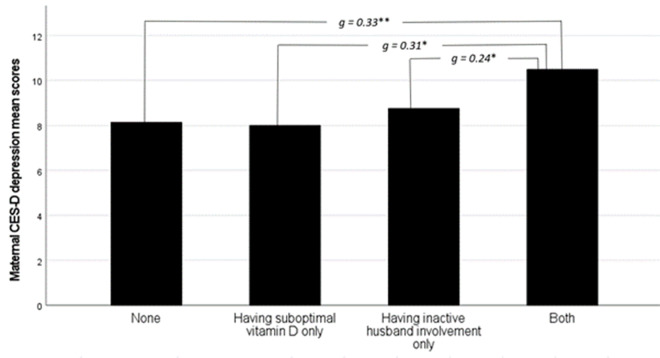
Group differences in maternal CES-D depression scores based on their vitamin D and husband involvement status. *Note*: Post-hoc multiple comparisons with Tukey’s HSD adjustment: *< 0.05; **< 0.001.

**Figure 2.**
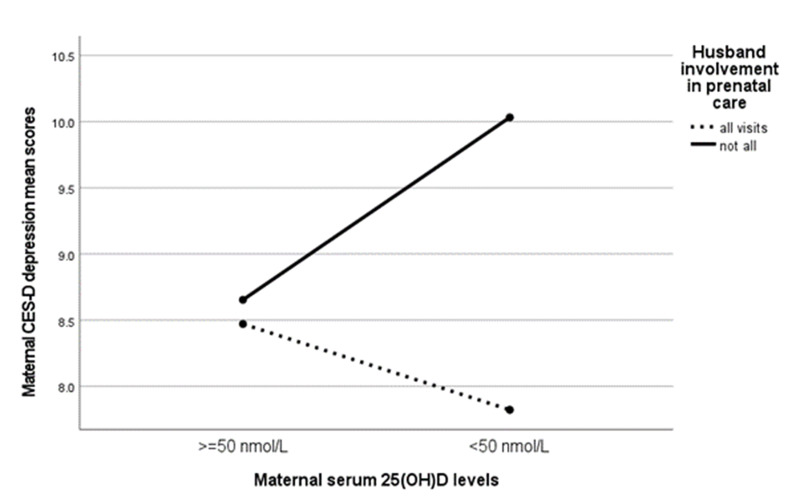
Husband involvement moderated effect of vitamin D status on the severity of depressive symptoms measured with CES-D self-report.

Figure 1 shows the differences in mean depression scores between the mother groups, categorized based on their vitamin D and husband involvement status. Mothers who lacked both optimal vitamin D and active husband involvement exhibited significantly higher levels of depressive symptoms compared to the other three groups. To test our primary hypothesis, we utilized regression models with a bootstrapping method to examine the crude and adjusted relationship between maternal 25(OH)D concentrations and depressive symptoms. Husband involvement was included as a moderator in both models. The coefficients for the models, along with their 95% confidence intervals, are presented in Table 2. Both crude and adjusted models were able to predict a significant, albeit small, amount of variance in the dependent variable (depression scores). Nonetheless, the association between husband involvement and maternal depression score was only significant in the crude model. After adjusting for the mother’s age, parity and socio-economic status, this association became nonsignificant, whereas the interaction between maternal 25(OH)D concentrations and husband involvement became significant. The results of simple slopes analysis showed that suboptimal 25(OH)D concentrations were associated with higher depression scores only among women whose husbands were less involved in antenatal care (β = 1.32, 95% CI = [0.22, 2.42], *p* = 0.019). These results allow us to accept the hypothesis, since the association between maternal 25(OH)D concentrations and depression scores was not significant for women whose husbands were actively involved in antenatal care. Figure 2 displays the graphical illustration of the relationship between maternal 25(OH)D concentrations and depression scores during pregnancy stratified by the level of husband’s involvement in antenatal care.

**Table 2.** Models predicting maternal depression scores based on their vitamin D status and husband involvement. Coefficients with 95% CI (in parenthesis below coefficient) are presented for each model

Crude model					Adjusted model^a^				
	β (95% CI)	*SE*	*T*	*P*		β (95% CI)	*SE*	*T*	*P*
Suboptimal vitamin D	−0.14 (−1.70, 1.42)	0.80	−0.18	0.861	Suboptimal vitamin D	−0.65 (−2.19, 0.90)	0.79	−0.82	0.412
Inactive husband involvement	0.62 (0.04, 1.19)	0.29	2.10	0.036	Inactive husband involvement	0.19 (−0.40, 0.76)	0.30	0.61	0.540
Vit D × husband involvement	1.88 (−0.03, 3.79)	0.97	1.93	0.054	Vit D × husband involvement	2.03 (0.14, 3.91)	0.96	2.11	0.035
*R*^2^	0.01				*R*^2^	0.03			
*F*(3, 2979)	5.81				*F*(3, 2975)	14.39			
*p*	<0.001				*p*	<0.001			

a Model adjusted for the mother’s age, parity, and socio-economic status

## Discussion

This study is the first, to our knowledge, to examine the association between vitamin D status and depressive symptoms in pregnant women as a function of their husband’s involvement in antenatal care. This work represents an important effort to combine biological and psychosocial perspectives in understanding the variations in the severity of depressive symptoms among pregnant women. The findings of this study can improve our understanding of depressive symptoms during pregnancy and emphasize the need for more interdisciplinary research and services in this area.

Through the analysis of maternal characteristics, we found that both income and parity were associated with maternal depressive symptoms and vitamin D status during pregnancy. Previous research has shown that having multiple childbirth experiences, coupled with inadequate vitamin D intake, can elevate the risk of vitamin D deficiency (Jeyakumar *et al.*, 2021). While the physical changes after each pregnancy are well-documented, the impact of parity on maternal depressive symptoms remains poorly understood. Our findings contribute to the existing literature by demonstrating that increased parity can correlate with poorer physical and mental well-being. Another notable finding is that although husband involvement has a protective effect on maternal depressive symptoms, its level tends to decline with each successive childbirth. This observation underscores the importance of reminding spouses and healthcare providers about the benefits of providing adequate support and care to pregnant women during each pregnancy, irrespective of their prior pregnancy and delivery experiences. Likewise, the availability of financial resources appeared to play a role in the vitamin D status and depressive symptoms of the women in this study. This echoes previous research linking socio-economic disadvantage to deficiencies in vitamin D and depression during pregnancy (Kiely *et al.*, 2020; Verbeek *et al.*, 2019). Targeted interventions that offer a combination of physical and mental health guidance and support could be particularly beneficial for women with lower levels of education and those with limited financial resources.

As expected, we did not find a link between the level of husband involvement in antenatal care and the vitamin D status of pregnant women. While the involvement of husbands may not be involved in the relationship between vitamin D status and depressive symptoms among pregnant women, it could be a contributing factor to individual differences in this relationship. Hence, this study also examined the role of husband involvement as a moderator of the link between vitamin D status and depressive symptoms in pregnant women. We compared women who had active participation from their husbands in antenatal care to those who did not. The findings confirmed our hypothesis, showing that pregnant women with both suboptimal 25(OH)D levels and inactive husband involvement had the highest levels of depressive symptoms and demonstrated large to medium-sized difference compared to other groups. On the other hand, when their husbands were actively engaged in antenatal care, their depressive symptoms did not differ by vitamin D status. Although there was no direct link between involving husbands in antenatal care and the mother’s vitamin D levels, husband involvement in antenatal care can empower mothers to cope with stress and discomfort during pregnancy. Research has demonstrated that partner engagement during pregnancy can yield favourable psychological outcomes, including a reduction in maternal depressive symptoms (Boekhorst *et al.*, 2019; Eddy and Fife, 2021). In contrast, solely prescribing dietary or supplementary interventions may not adequately address the emotional distress caused by the lack of partner involvement in antenatal care. Therefore, interdisciplinary interventions should be implemented to assist vulnerable pregnant women in expanding their social networks, securing more support sources and maintaining a balanced diet. This will give them more options to meet their psychological, social, and nutritional needs.

This study presents several merits, including the recruitment of a sizable cohort and the implementation of cross-domain assessments to elucidate depression. Nevertheless, caution should be exercised in interpreting the findings due to the study’s limitations. Firstly, the interaction between maternal vitamin D status and husband involvement was found to correlate with maternal depressive symptoms during pregnancy. However, the overall predictive capacity of the regression model was limited, indicating that additional factors not considered in this study may also contribute to variations in depressive symptoms. One such factor is insufficient iodine intake during pregnancy, which has been associated with higher levels of depressive symptoms in recent research (Brantsæter *et al.*, 2022). It is probable that vitamin D is just one of many nutrients that play a role in depression development. Future research should explore whether the dietary profile is more critical than individual nutrients in predicting depressive trends. Secondly, as the participants were exclusively married, the findings may not be generalizable to unmarried women who may have dissimilar expectations regarding their partner’s involvement in pregnancy. Furthermore, all participants in this study were in the advanced stages of pregnancy (25–35 weeks). Their views on the significance of involving husbands in antenatal care may differ from those in earlier stages of pregnancy. Future studies should examine the influence of marital status and fluctuations in husband involvement across various stages of pregnancy and their potential impact on a woman’s depressive symptoms over time. Finally, in this study, active husband involvement was defined as the husband accompanying the mother to every antenatal care visit. Since the quality of care and support provided by the husband is associated with maternal health and well-being (Eddy and Fife, 2021), future studies should explore various dimensions of husband involvement, beyond mere attendance, to determine their impact on maternal depressive symptoms during pregnancy.

In conclusion, our study highlights the multifaceted nature of depression during pregnancy, with contributions from both biological and psychosocial factors. Specifically, women who exhibit low levels of vitamin D and receive inadequate support from their spouses are at greater risk of experiencing depression. It is imperative to offer tailored interventions that address the emotional well-being and nutritional status of these vulnerable mothers during pregnancy. Furthermore, future research ought to examine other factors like social support from friends and physical health issues that might also impact maternal depression during and after pregnancy, beyond the variables examined in this study.

## Data Availability

The data that support the findings of this study are available on request from the corresponding author.
